# Trade-offs constrain adaptive pathways to the type VI secretion system survival

**DOI:** 10.1016/j.isci.2023.108332

**Published:** 2023-10-26

**Authors:** Kathryn A. MacGillivray, Siu Lung Ng, Sophia Wiesenfeld, Randi L. Guest, Tahrima Jubery, Thomas J. Silhavy, William C. Ratcliff, Brian K. Hammer

**Affiliations:** 1 School of Biological Sciences, Georgia Institute of Technology, Atlanta, GA, USA; 2 Parker H. Petit Institute for Bioengineering & Bioscience, Georgia Institute of Technology, Atlanta, GA, USA; 3 Center for Microbial Dynamics and Infection, Georgia Institute of Technology, Atlanta, GA, USA; 4 Department of Molecular Biology, Princeton University, Princeton, NJ, USA

**Keywords:** Bacteriology, Biological sciences, Evolutionary mechanisms

## Abstract

The Type VI Secretion System (T6SS) is a nano-harpoon used by many bacteria to inject toxins into neighboring cells. While much is understood about mechanisms of T6SS-mediated toxicity, less is known about the ways that competitors can defend themselves against this attack, especially in the absence of their own T6SS. Here we subjected eight replicate populations of *Escherichia coli* to T6SS attack by *Vibrio cholerae*. Over ∼500 generations of competition, isolates of the *E. coli* populations evolved to survive T6SS attack an average of 27-fold better, through two convergently evolved pathways: *apaH* was mutated in six of the eight replicate populations, while the other two populations each had mutations in both *yejM* and *yjeP*. However, the mutations we identified are pleiotropic, reducing cellular growth rates, and increasing susceptibility to antibiotics and elevated pH. These trade-offs help us understand how the T6SS shapes the evolution of bacterial interactions.

## Highlights

• *E. coli* evolved 27-fold higher resistance to *V. cholerae* T6SS after 500 generations
• Convergent evolution reveals just mutational pathways for improving T6SS survival
• A mechanosensitive channel gene mutation provides broad spectrum resistance to T6SS
• Mutations improving T6SS survival reduce growth, revealing an evolutionary tradeoff

## Graphical abstract

**Figure:**
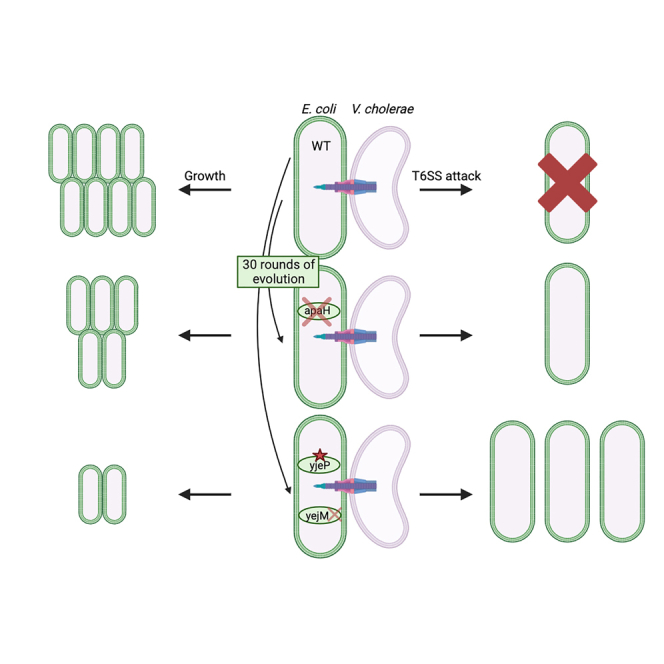

## Introduction

Bacteria are one of the most common forms of life on Earth and often live in polymicrobial biofilms. Within this complex community, negative bacterial interactions are common,^1^ due to intense competition for resources such as nutrients and space. One way for bacteria to gain an advantage over their competitors is by killing them. They have evolved two major classes of antagonistic mechanisms to eliminate competitors: diffusible and contact-dependent. Diffusible antibacterial molecules have been extensively described in soil bacteria such as *Streptomyces*, which produces antibiotics (e.g., streptomycin, kanamycin, and tetracycline) to kill competitors, gain resources, and maintain symbiosis with associated plants.^2^ *Pseudomonas aeruginosa* is also known to secrete lethal toxins such as pyocyanin, exotoxin A, and ExoU that aid in competing against other microbes and targeting human cells during infections.^3^^,^^4^^,^^5^ On the other hand, contact-dependent antagonisms are less diverse and their social dynamics remain relatively understudied.^6^ One noteworthy counterexample is the Type VI secretion system (T6SS), discovered in 2006. The T6SS is a contact-dependent “nano-harpoon” that kills neighboring cells by injecting them with a set of toxic proteins.^7^ The T6SS is estimated to be found in ∼25% of all Gram-negative bacterial species,^8^ and targets diverse cell types, including eukaryotes such as macrophages, and both Gram-positive and Gram-negative bacteria such as *Escherichia coli*, in both an environmental and host context.^7^^,^^9^^,^^10^^,^^11^

While the regulation, genetics and functional mechanics of the T6SS have been well studied,^12^^,^^13^^,^^14^ we know less about how targeted cells respond, defend, and survive T6SS attack.^15^^,^^16^^,^^17^^,^^18^ Similar to antibiotic resistance, one strategy is to neutralize the toxins. Bacteria wielding a T6SS that carries anti-microbial toxins do not intoxicate themselves or their sibling cells because a conjugate immunity protein is encoded in the same gene cluster as each toxic effector protein.^19^^,^^20^^,^^21^ However, cells lacking immunity proteins are vulnerable to the toxins. In some cases, bacteria can acquire a library of orphan immunity proteins via horizontal gene transfer and mobile genetic elements, enabling them to survive toxins expressed by unrelated cells.^22^^,^^23^^,^^24^^,^^25^ *P. aeruginosa,* a model organism for T6SS research, has evolved particularly sophisticated regulation of its T6SS. Cells that have been provoked by the T6SS of another bacterium can assemble their own T6SS apparatus and launch a counter-attack in the direction from which the first attack came.^26^ *P. aeruginosa* is additionally able to induce T6SS attack in response to kin cell lysis, via a mechanism called “danger sensing.”^27^ Physical processes can also offer protection. Extracellular polysaccharide can protect cells from T6SS attack, as does the accumulation of cellular material from lysed cells and physical separation, which are both consequences of T6SS antagonism.^28^^,^^29^^,^^30^^,^^31^^,^^32^ External signaling can play a role in this protection, with recent reports that the presence of glucose enhances survival of *E. coli* cells to T6SS attack, mediated through cyclic AMP and its cognate target, the CRP regulator.^33^ Other regulators that coordinate stress response systems, such as Rcs and BaeSR may also play an important role, as deletions of these genes reduce survival from attack.^34^^,^^35^ Transposon sequencing (Tn-seq)^36^ offers one approach to identify genes that affect T6SS resistance, uncovering mutations that either increase or decrease survival.^37^ However, this technique has a limited range of mutations it can uncover, identifying only single null mutations contributing to a phenotype, but not deleterious mutations in essential genes, functional point mutations, or epistatic relations between multiple genes. Mutagenic screens also do not take pleiotropic side effects of mutations into account. For example, mutations that increase T6SS survival but come at a steep cost to cellular growth rates would be detected in such a screen but might not be expected to arise under conditions where reproductive fitness is important.

Experimental evolution^38^ circumvents many of these issues, allowing the interrogation of the whole genome in a high-throughput, unbiased manner. By including periods of growth between rounds of T6SS attack, this approach allows selection to include key pleiotropic fitness effects. Clonal interference among beneficial mutations means that only a small fraction of possible beneficial mutations will arise to high frequency in any given experiment,^39^ typically favoring those that are most adaptive. Rather than reporting all possible routes to surviving T6SS attack, experimental evolution thus provides insight into genetic mechanisms that provide the largest fitness advantage over hundreds of generations of growth and periodic T6SS assault.

In this article, we explore how *E. coli* evolves resistance to T6SS attack by *Vibrio cholerae*. After ∼500 generations of growth, punctuated by 30 rounds of attack by the T6SS, we identified two main mutational pathways, each of which convergently evolved in multiple populations, that enabled dramatically improved survival by *E. coli* during T6SS attack. Similar to antibiotic resistance,^40^ we find that there was a strong trade-off between increased T6SS survival and reduced fitness during growth, which may help explain the continued efficacy of T6SS effectors in natural populations despite billions of generations of T6SS exposure.

## Results

### Experimental evolution of the type VI secretion system resistance

**Figure 1:**
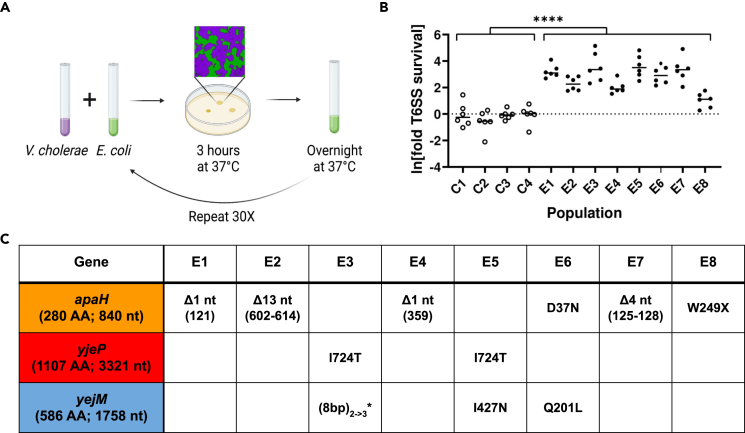
Experimental evolution of resistance to *V*. *cholerae*’s Type VI Secretion System (A) Experimental design. We experimentally evolved eight replicate populations of *E. coli*. Each round of selection included ∼16 generations of growth in liquid media, followed by co-culture with T6SS-expressing *V. cholerae* on solid media, where initially the vast majority of *E. coli* were killed. *V. cholerae* were removed via antibiotics, and the surviving *E. coli* resumed growth in liquid media. (B) Over 30 rounds of selection, *E. coli* in the T6SS treatment evolved a 27-fold increase in T6SS survival relative to the ancestral *E. coli* strain, while controls competed against a T6SS(−) *V. cholerae* did not evolve a significant increase in T6SS resistance. ∗∗∗∗ denotes a difference in survival with p ≤ 0.0001, determined via ANOVA and a pre-planned contrast. (C) Convergent evolution of genes affording T6SS survival. Three genes were mutated in all eight independently evolving populations: *apaH* arose in six, while mutations in *yejM* and *yjeP* arose in the other two populations. For deletions (Δ), numbers in parentheses refer to the nt position of the deletion. (8nt)_2->3_∗ refers to an 8 nt repeat that expanded from 2 repeats to 3 repeats long, resulting in a frameshift mutation. W249X refers to a premature stop codon at position 249, resulting in a protein product truncated near the C terminus. (AA = amino acids; nt = nucleotides).

We report the development of an experimental evolution platform with two model organisms, to identify mechanisms by which bacteria can become resistant to T6SS attack (Figure 1A). We experimentally evolved eight replicate populations of *E. coli* MG1655, exposing them to daily attack by a *V. cholerae* C6706 strain variant that constitutively expresses the building blocks of the apparatus and its four T6SS effectors, two that act in the periplasm to degrade the peptidoglycan cell wall (VgrG3 and TseH) and two that disrupt membranes (TseL and VasX) (see STAR Methods).^21^^,^^41^^,^^42^^,^^43^ The two species were co-cultured on agar plates in 10:1 ratio (killer:target) to ensure direct contact between cells, which is necessary for T6SS attack. 99.99% of our *E. coli* ancestor were killed by *V. cholerae* during the solid-media killing phase of the experiment, imposing strong selection for T6SS survival. Between rounds of competition, *E. coli* populations were grown for ∼16 generations in LB medium overnight, in the presence of kanamycin and chloramphenicol to prevent *V. cholerae* growth. We also evolved four control populations, competing the same ancestral *E. coli* against a T6SS-deficient *V. cholerae ΔvasK* strain. We reasoned that mutations arising in these four control populations would account for adaptation in our environment, including growth, dilution, and co-culture with *V. cholerae* on solid media, but not from injury from the T6SS. After 30 rounds of selection, evolved strains were an average of ∼27-fold more resistant to *V. cholerae*’s T6SS attack, and the control populations had on average 3.9% higher survival, a negligible difference (*F*_*11,71*_ = 15.8, p *≤* 0.0001, ANOVA with replicate nested in treatment. Fold survival was log-transformed prior to analysis to homogenize variances, and treatment effect was assessed with pre-planned contrast, *F*_*1,60*_ = 234, p ≤ 0.0001; Figure 1B).

### Identifying and characterizing key mutations

We identified mutations arising in our experiment by sequencing a single genotype from each population after 30 rounds of selection. With an average of 2.75 (standard deviation 1.09) mutations per genome in the experimentally evolved isolates of each population, we chose to focus on mutations that occurred in more than one replicate population, as convergent evolution strongly suggests these mutations are adaptive (Figure 1C; Figure S1). Six of the eight isolates had mutations in *apaH*. Four of which are frameshift mutations, suggesting they resulted in loss-of-function (Figure 1C). This gene is responsible for the “de-capping” of mRNAs in a bacterial cell.^44^ Little is known about the global regulatory effect of loss of *apaH*, but it is hypothesized that a null mutation leads to RNA stabilization. Notably, the isolate from population E8 only gained a ∼3-fold increase in survival relative to its ancestor; which was significantly lower than five of the seven other replicate experimental populations (fold survival was log-transformed prior to analysis to homogenize variances, pairwise differences between each replicate population assessed via ANOVA and Tukey’s HSD with overall significance at *α* = 0.05; Figure 1B). The mutation in *apaH* found in this isolate creates a premature stop codon near the end of the gene (amino acid 249 out of 280) that likely retains the partial function of *apaH*, resulting in a more modest survival advantage.

Two of the eight isolates did not have a mutation in *apaH*. Instead, these two populations each had missense or frameshift mutations in both *yjeP* (also known as *mscM*) and *yejM*, suggesting an interaction between these two genes (Figure 1C). *yjeP* encodes a mechanosensitive channel that protects cells from osmotic shock.^45^ The gene *yejM* (also known as *pbgA* and *lapC*) encodes a metalloprotein that regulates bacterial lipopolysaccharides biosynthesis.^46^^,^^47^ Deletion of *yejM* is lethal in *E. coli*, while C-terminal truncation mutations result in the partial function of the gene.^48^ Both mutations we found in *yejM* occur near the C-terminus.

**Figure 2:**
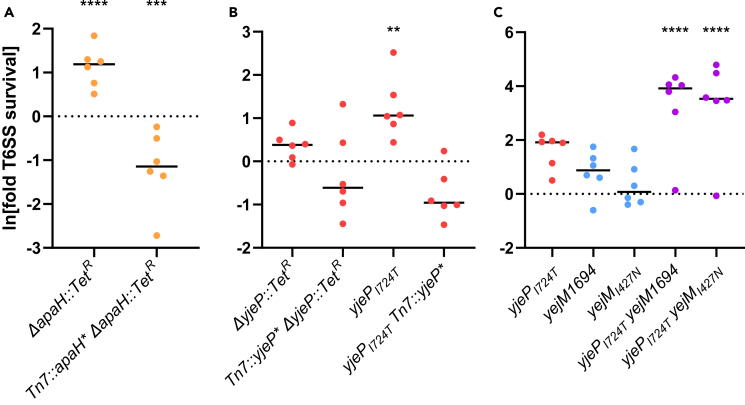
While all mutations of interest increase T6SS resistance in various degrees, the *yjeP/yejM* double mutants survive significantly better (A) *E. coli* with the deletion of *apaH* or (B) *yjeP*_*I724T*_ mutation had a slight increase in T6SS resistance relative to the ancestral *E. coli* strain, which was not observed in the other variants. (C) The combination of *yjeP*_*I724T*_ and mutations in the C-terminus of YejM significantly improved the *E. coli* survival by more than 42-fold. Linked markers used to construct the mutants are not indicated in the figure. ∗∗∗∗, ∗∗∗, and ∗∗ denote differences in survival with p ≤ 0.0001, p ≤ 0.001, and p ≤ 0.01 respectively, determined via ANOVA and Dunnett’s Multiple Comparison.

To test the function of mutations found in *apaH*, *yjeP,* and *yejM* independent of the role of other mutations that arose in experimental lineages (Figure S1), we re-engineered mutations in these genes in the ancestral strain. A clean deletion of *apaH* increases T6SS protection by 3-fold, whereas *E. coli* carrying a single copy of *apaH* expressed from a heterologous constitutive promoter at the Tn7 site is 0.4-fold more susceptible than the ancestor (fold survival was log-transformed prior to analysis to homogenize variances, comparison of means was assessed with one-sample *t*-test (*μ* = 0) and Bonferroni correction with overall significance at *α* = 0.05, p ≤ 0.0001 and p ≤ 0.001; Figure 2A; see STAR Methods).

### *yjeP*_I724T_ is a gain-of-function mutation that confers the type VI secretion system resistance

*yjeP* is one of the four paralogs predicted to encode the MscS mechanosensory channel.^45^ An identical missense mutation in *yjeP* (*yjeP*_*I724T*_) occurred independently in two lineages (Figure 1C), suggesting that this amino acid substitution enhances T6SS survival and represents a gain-of-function mutation. In the ancestor genetic background, we introduced a *yjeP* disruption*,* constitutively expressed *yjeP,* and reconstructed the *yjeP*_*I724T*_ mutation. Interestingly, neither the absence of *yjeP* nor its constitutive expression affected T6SS survival. However, *E. coli* carrying the *yjeP*_*I724T*_ mutation experienced a ∼4-fold survival benefit (fold survival was log-transformed prior to analysis to homogenize variances, pairwise differences between each replicate population assessed via ANOVA and Dunnett’s test with overall significance at *α* = 0.05, p ≤ 0.01; Figure 2B).

**Figure 3:**
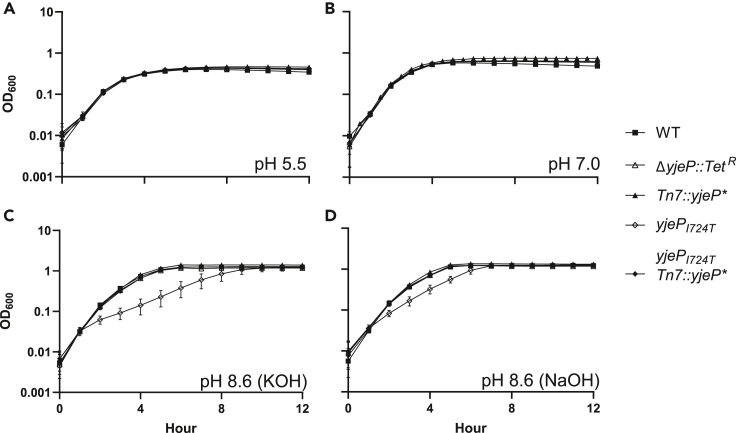
*E*. *coli yjeP*_*I724T*_ has reduced fitness under basic conditions *E. coli* and its *yjeP* derivatives grow similarly under acidic (A) and neutral pH. (B) In basic media adjusted with either KOH (C) or NaOH (D), however, the *yjeP*_*I724T*_ mutant has a significant decrease in division rate. Linked markers used to construct the mutants do not affect growth in the tested conditions (Figure S8). To determine if different genotypes grow at different rates as a function of pH, we examined log-transformed (with a constant of 1 added to each OD value prior to transformation to maintain positive values) OD over the first four hours of growth via ANCOVA. The relative growth rate of Δ*yjeP*::I724T, as measured by the interaction between Ln(OD) and strain, and confirmed by a Tukey's HSD post hoc test, was highly significant in alkaline conditions (pH 8.6), both when adjusted with KOH (F(4, 260) = 66.51, p < 0.0001) and NaOH (F(4, 260) = 35.22, p < 0.0001). In contrast, at pH 5.5 (F(4, 260) = 0.55, p = 0.7) and pH 7 (F(4, 260) = 1.53, p = 0.193), the interactions were not significant, suggesting that at these lower pHs all strains grew at the same rate.

Because YjeP is predicted to be a mechanosensitive channel,^45^ we determined how the *yjeP*_*I724T*_ mutant responded to pH and osmotic shock, classic stressors for probing mechanosensor function. A *yjeP* null mutant (Δ*yjeP*) and a mutant expressing *yjeP* constitutitvely (*yjeP*∗) behaved like WT Figure 3). Interestingly, while the *yjeP*_*I724T*_ mutant was unaffected by changes in osmolarity (Figures 3A and 3B), it did exhibit highly significant decreases in maximum growth rate in the exponential phase when grown in alkaline conditions (Figures 3C and 3D), suggesting that YjeP may be an ion channel (OD_600_ was log-transformed prior to analysis, and the growth rates were assessed with linear regression comparison of the slopes in the exponential phase with significance as detailed in the legend of Figure 3). To determine whether YjeP is the only MscS mechanosensitive channel protein that can affect T6SS resistance, we also tested one of the three YjeP homologs, YbdG,^45^ because a prior study showed a *ybdG*_*I176T*_ gain-of-function mutation also confers sensitivity to osmotic shock.^49^ Unlike *yjeP*_*I724T*_, the *ybdG*_*I167T*_ did not confer T6SS resistance, nor did a *ybdG* null (Figure 3; Figure S7). Thus, we conclude that *yjeP*_*I724T*_ is a gain-of-function, or co-dominant, mutation in an ion channel that confers T6SS resistance.

### *E. coli yjeP/yejM* double mutants are much more resistant to diverse the type VI secretion system toxins

The fact that *yejM* and *yjeP* accrued mutations in parallel in two independent populations suggests there may be an epistatic relationship between these two mutations. To test this hypothesis, we introduced both *yejM* mutations into the ancestral *E. coli* without and with the *yjeP*_*I724T*_ mutation. While the *yjeP*_*I724T*_ mutation confers a modest benefit (4-fold increased survival; fold survival was log-transformed prior to analysis to homogenize variances, pairwise differences between each replicate population assessed via ANOVA and Dunnett’s test with overall significance at *α* = 0.05, p ≤ 0.01; Figure 2B), the presence of either *yejM* mutation by itself had no effect on resistance (Figure 2C). However, the *yjeP*_*I724T*_ mutation combined with either *yejM* mutation enables a ∼40- to 50-fold increase in survival compared to the ancestor (fold survival was log-transformed prior to analysis to homogenize variances, pairwise differences between each replicate population assessed via ANOVA and Dunnett’s multiple comparison with overall significance at *α* = 0.05, p ≤ 0.0001; Figure 2C). In other words, mutation in *yejM* increases resistance only in strains that also have the *yjeP* point mutation.

**Figure 4:**
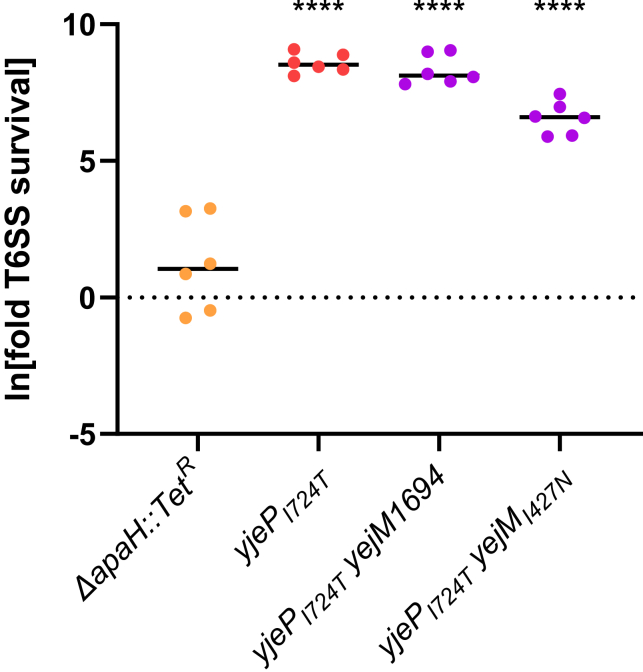
The *yjeP* and the *yjeP/yejM* mutants provide general resistance to T6SS attack When competed against *V. cholerae* with a set of toxins not encountered during experimental evolution, *E. coli* mutants with *yjeP*_*I724T*_ had an increase in T6SS resistance relative the ancestral *E. coli* strain, whereas the deletion of *apaH* did not provide protection against T6SS effectors not encountered prior. Linked markers used to construct the mutants are not indicated in the figure. ∗∗∗∗ denotes a difference in survival with p ≤ 0.0001, determined via ANOVA and Dunnett’s Multiple Comparison.

We next examined whether the mutations that arose in our experiment provide general resistance to T6SS attack, or are specific to the toxins employed by the *V. cholerae* C6706 strain, used in this evolution screen, which codes three auxiliary T6SS effectors in addition to the large cluster. We therefore competed each mutant *E. coli* strain against an environmental isolate of *V. cholerae* killer*,* BGT41 (also known as VC22), which encodes a constitutive T6SS with effectors predicted to have enzymatic activities distinct from those produced by C6706 and encountered by *E. coli* during experimental evolution.^50^^,^^51^ This environmental isolate is a superior killer of *E. coli*, relative to C6706,^51^ necessitating that we perform our killing assays at a 1:4 killer:target ratio, rather than the 10:1 ratio used with C6706 (at the original 10:1 ratio, no *E. coli* survived). This change in ratio resulted in different values for fold-changes in survival compared to earlier plots. However, the relative survival increases of the *yjeP*_I724T_ mutants rather than the value of the fold-increase, demonstrate general resistance. Evolved strains with *yjeP*_*I724T*_ and *yjeP*/*yejM* double mutations survived significantly better than the *E. coli* ancestor, but *apaH* did not measurably increase survival (fold survival was log-transformed prior to analysis to homogenize variances, pairwise differences between each replicate population assessed via ANOVA and Dunnett’s multiple comparison with overall significance at *α* = 0.05, p ≤ 0.0001; Figure 4). In addition, unlike with the C6706 killer (Figure 2C), the *yejM* mutations did not further increase the survival of the *yjeP*_*I724T*_ mutant (Figure 4). This suggests that I724T in *yjeP* may provide broad spectrum resistance to T6SS while protection conferred by mutations in the YejM C-terminus and in *apaH* may depend on the specific effector employed.

### Two evolutionary pathways to the type VI secretion system resistance

**Figure 5:**
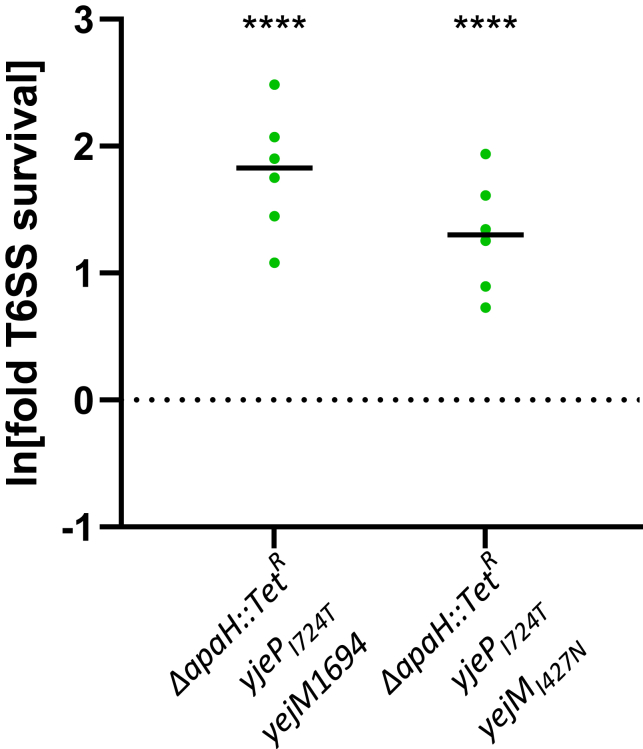
Triple mutants are more resistant to T6SS attack than double mutants The addition of an *apaH* disruption further increases resistance of strains that already have the *yjeP*_*I724T*_*/yejM* C-terminal mutation genotype. Relative survival is shown for triple mutants with each type of *yejM* mutation. Values are shown as fold-increases relative to the corresponding double mutants lacking the *apaH* disruption. Linked markers used to construct the mutants are not indicated in the figure. ∗∗∗∗ denotes a difference in survival with p ≤ 0.0001, comparison of means was assessed with ANOVA and Dunnett’s Multiple Comparison (*μ* = 0) with overall significance at *α* = 0.05.

Our results suggest two distinct mutational pathways lead to increased T6SS survival in *E. coli*. To test whether these pathways are independent of one another, or are instead mechanistically redundant, we constructed *E. coli* strains with the *apaH* disruption, *yjeP*_*I724T*_, and either *yejM*(8bp+) or *yejM*_*I427N*_. We refer to these strains as the “triple mutants.” We compared each of the two triple mutants to their corresponding double mutants containing only *yjeP*_*I724T*_ and one of the *yejM* C-terminal mutations. In other words, we sought to determine whether the deletion of *apaH* would further increase the resistance of the *yejM/yjeP* mutants, which survive the best in response to T6SS attack of our reconstructed strains. Both triple mutants survived T6SS attack an average of 7.5- and 4.5-fold better than their respective double mutants (or 111- and 66-fold better than the ancestor; Figure 5; comparison of means was assessed with one-sample *t*-test (*μ* = 0) and Bonferroni correction with overall significance at *α* = 0.05, p ≤ 0.0001, see STAR Methods) We therefore conclude that *apaH* disruption and *yjeP/yejM* mutation represent two mechanistically distinct evolutionary pathways to T6SS resistance.

### Experimental evolution reveals a trade-off between the type VI secretion system resistance and growth rate

**Figure 6:**
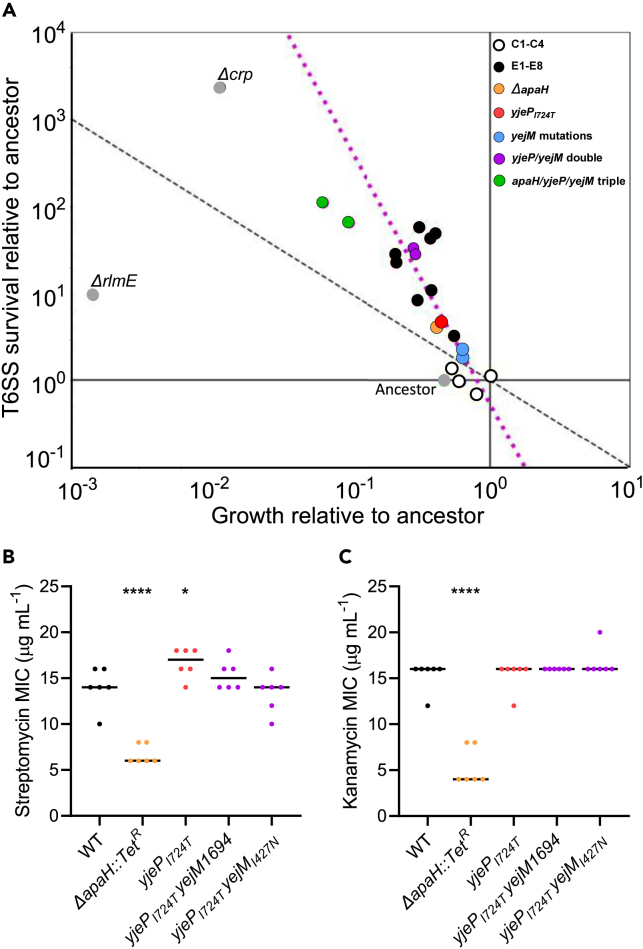
Trade-offs between T6SS resistance and fitness during growth (A) Mutations conferring a larger T6SS survival advantage also resulted in a greater reduction to reproductive fitness. Plotted are the change in frequency of each mutant across one 16 generation growth assay, and one T6SS attack, following the protocols from our evolution experiment. The dashed gray line represents a fitness isocline, y=1/x, where fitness across one complete round of growth and T6SS survival selection is equal to that of the ancestor. In other words, the isocline represents where increased fitness during T6SS survival is exactly outweighed by decreased fitness in the growth phase. The dashed pink line represents a simple linear regression highlighting the trade-off between increased survival and decreased growth rate over 500 generations of evolution, as well as strains engineered with key mutations; log_10_(*survival*) *=* −2.9 ·log_10_(*growth*) - 0.27, *r*^2^ = 0.65, p = 3.01·10^−5^. This regression does not include the triple mutants, or the Δ*crp* and Δ*rmlE* mutants, which did not occur in our experiment. (B and C) Disruption of *apaH* results in decreased MIC for streptomycin (B) and kanamycin (C). The point mutation *yjeP*_*I724T*_ does not affect susceptibility to these antibiotics. Linked markers used to construct the mutants are not indicated in the figure. ∗∗∗∗ and ∗ denote differences in survival with p ≤ 0.0001 and p ≤ 0.05, determined via ANOVA and Dunnett’s Multiple Comparison test.

So far we have shown that *E. coli* readily evolves resistance to T6SS attack, one of the most common mechanisms of antimicrobial warfare. Why, after billions of years of evolution, are bacteria still so poorly defended against the T6SS? Evolutionary theory predicts that trade-offs between antibiotic resistance and other fitness-dependent traits can maintain susceptibility.^52^ To test this hypothesis, we examined the effect of each mutation on cellular growth rate by competing them against the ancestral genotype of *E. coli*, under the conditions that mirrored our selection experiment. Mutations in *apaH*, *yejM*, and *yjeP* decreased fitness during growth (Figure 6). In fact, there was an overall negative correlation between T6SS survival and growth rate for the strains generated in this study (log_10_(*survival*) *=* −2.9 log_10_(*growth*) - 0.27, *R*^*2*^ = 0.63, p = 2.34·10^−5^; this regression excludes the *crp* and *rlmE* mutants (described later in discussion), as well as the triple mutants (green dots) that never arose during experimental evolution; Figure 6A).

Our evolution experiment consisted of ∼16 generations of exponential growth in LB media each day, followed by a round of T6SS killing on plates. We thus calculated a fitness isocline across the phase space of this trade-off (black dashed line in Figure 6A), along which a mutant would have equal fitness to the ancestor across one round of growth and killing, with the equation y=1/x (in log_10_ space). For example, along this line, a 100-fold increase in T6SS survival is exactly canceled out by a 100-fold decrease in overnight growth. Mutations that lie above this line should be more fit than our ancestral strain, while mutations below the line should be maladaptive. Perhaps unsurprisingly, given the strong selection on both growth and T6SS survival, all mutations (and combinations) we identified in evolved isolates are adaptive. The triple mutants (*yjeP/yjeM/apaH*) suffer a growth defect strong enough to place them below the pink regression line where evolved isolates and reconstructed mutants lie. Because the triple mutants lie above the black fitness isocline, they are more fit in this fluctuating growth/attack cycle than the ancestor; however, they are less fit than the trend shown by the evolved isolates and other reconstructed mutants. This may explain why, despite a high degree of convergent evolution in our overall experiment, the combination of all three mutations was not seen in the sequenced isolates.

We also measured growth and survival rates for two mutants that did not arise in our experimentally evolved populations – strains with disruptive mutations in *crp* and *rlmE*. We have previously shown that the deletion of *crp*, a global transcriptional repressor, results in increased survival to the T6SS in *E. coli*, but also greatly reduces growth rate.^33^^,^^53^^,^^54^ While this mutation does fall above the fitness isocline, it did not appear in our evolution experiment (Figure 6). This could be because the decreased growth rate is too severe for the increase in T6SS resistance to be worth it. Because all of our mutations of interest result in decreased growth rate, we also sought to test whether decreased growth rate was sufficient to increase T6SS resistance. For example, slower growth could prevent microcolonies of the two strains from physical contact on the plate during the course of the co-culture competition. We engineered a slow-growing strain of *E. coli* by deleting *rlmE*, which encodes for a methyltransferase that modifies the ribosomal RNA (rRNA) - an essential process for efficient protein synthesis. As a result of this deletion, the strain’s growth was significantly hampered, amounting to just 0.14% of its unaltered ancestor’s growth over a single cycle (two-sided *t*-test, p = 8.75·10^−5^). This strain results in much smaller colonies when growing on plates, but has only a 10-fold increase in survival when challenged with T6SS attack (Figure S9). The *rlmE* mutant is below the fitness isocline in Figure 6A, as the modest increase in survival is not commensurate with the massive growth defect of this mutant. This shows that slower growth is a side effect of mutations that increase T6SS resistance, not a cause of increased resistance.

Another trade-off we tested is susceptibility to aminoglycoside antibiotics. The *apaH* disruption strain has a significantly lower minimum inhibitory concentration (MIC) than the ancestor when grown in streptomycin and kanamycin (pairwise differences between each replicate population assessed via ANOVA and Dunnett’s test with overall significance at *α* = 0.05, p ≤ 0.0001 and p ≤ 0.05; Figures 6B and 6C), meaning that they are more susceptible to these antibiotics. This is consistent with previous work on *apaH*.^55^ However, strains containing the *yjeP* point mutation did not show increased susceptibility. For our evolution experiment, we used *E. coli* with two antibiotic resistance genes (chloramphenicol and kanamycin), allowing us to use these antibiotics to remove all *V. cholerae* from the population each day after the killing phase of the experiment. Given that *apaH* disruptions result in increased kanamycin susceptibility, it is possible that the *apaH* and the kanamycin resistance genes share gene regulatory interactions. Inclusion of plasmid-encoded kanamycin resistance and kanamycin in the media may have eased the growth tradeoffs incurred by *apaH* mutations.

## Discussion

In this article, we use experimental evolution to examine how bacteria adapt to frequent T6SS exposure. We subjected populations of *E. coli* to alternating selection for rapid growth followed by attack by *V. cholerae*’s T6SS (Figure 1A). All replicate populations evolving increased T6SS resistance (seven of the eight populations) utilized one of the two pathways: either a loss-of-function mutation in *apaH*; or a gain-of-function mutation I724T in *yjeP* combined with a partial loss-of-function in *yejM*, with both mutations necessary to provide a large survival advantage (Figures 1B and 1C). For a *yjeP*_*I724T*_ mutant, the protection appears to be broad-spectrum, increasing resistance, by more than 3000-fold, to effector proteins not previously encountered in the experiment (Figure 4). Interestingly, the *yjeP*/*yejM* double mutants are also comparatively resistant to T6SS attack when competing against *V. cholerae* BGT41 (Figure 4), suggesting *yjeP*_*I724T*_ provides a broader protection while the additional yejM mutations are specific to C6706 T6SS effectors (Figure 2C). While the mechanism underpinning this strain-specific effect is beyond the scope of this study, we hypothesize *yejM*_*I427N*_ still encodes a partially functional YejM periplasmic domain, whereas an insertion of 8 nt (*yejM*1694) results in a frameshift mutation, resulting in a complete disruption of the C-terminus.^56^ In *Salmonella enterica* and *E. coli,* truncation of the C-terminus was shown to disrupt the function of YejM, negatively impacting lipopolysaccharide biosynthesis. This leads to a defective outer membrane that leaks periplasmic proteins into the extracellular space.^57^ Periplasmic leakage may reduce the concentration of membrane-localized T6SS toxins injected into *E. coli* bearing the *yejM*1694 mutation, reducing their lethality. In contrast, mutations in *apaH* were specific to the T6SS effectors they were evolved against, showing no efficacy against a different strain of *V. cholerae* with T6SS effectors previously not encountered (Figures 2A and 4).

Of the two primary mutational pathways we focused on in this study, it is interesting that the less beneficial path to T6SS resistance, loss of function in *apaH,* evolved more times than the far more beneficial combination of *yjeP*_*I724T*_ and a partial loss-of-function of *yejM* (Figure 4). This is likely because it is easier to gain beneficial mutations in the *apaH* pathway: any loss of function mutation in the gene gives the phenotype, whereas the *yejM/yjeP* pathway requires more constrained mutations in two separate genes. The convergent evolution we observed in our experiment (identical *yjeP* SNPs in both populations evolving resistance via this mechanism) further suggests that specific mutations, not simple loss of function mutations, may be required in *yjeP.* Given the difference in T6SS resistance between evolved isolates with an *apaH* mutation (Figures 1B and 1C) and the constructed *apaH* mutant (Figure 2A), we hypothesize that other mutations acquired by the evolved populations may also contribute to T6SS survival.

Over 500 generations of experimental evolution in 8 replicate populations, we found just two pathways to increased T6SS resistance. The engineered triple mutants in both pathways confirm these two pathways are independent since both showed enhanced resistance relative to mutants in only a single pathway (Figure 5). While prior work has shown that many genes that can affect T6SS survival,^34^^,^^37^^,^^58^^,^^59^^,^^60^ implying that adaptation might be idiosyncratic among independent populations, our results suggest that adaptive routes to T6SS resistance are remarkably constrained. One possibility is that our populations are mutationally limited. This is unlikely, as we can expect ∼9.2 x 10^5^ mutations to arise within each growth cycle (based on ∼10^10^ cells being produced per cycle, a mutation rate of ∼0.2 x 10^−10^ per generation per base^61^ and a genome size of 4.6 MB), or 2.8 x 10^7^ mutations in each population over the course of the experiment. Instead, the high degree of evolutionary convergence in our experiment suggests that there may simply be relatively few routes to increased T6SS survival in which the benefits of the mutation, integrated across the culture cycle to include pleiotropic costs, are great enough to drive the clonal lineage to high frequency.

The evolution of resistance to diffusible antibiotics is well studied, with much attention directed toward adaptations to antibiotic drugs used in clinical setting.^62^^,^^63^^,^^64^ While the details depend on taxon and environment,^64^^,^^65^ antibiotic resistance often comes with trade-offs to other fitness components,^65^^,^^66^^,^^67^^,^^68^ although this is not always observed.^69^ Trade-offs are often noted in regard to mutations in essential genes that are targeted by antibiotic drugs, such as genes encoding ribosomal proteins.^70^ Compensatory evolution can often reduce initially severe costs of resistance, either via the fixation of epistatic mutations elsewhere in the genome, or by replacing initially costly resistance mutations with lower-cost alternatives.^70^^,^^71^^,^^72^^,^^73^ In contrast to diffusible antibiotics, the eco-evolutionary dynamics of contact-mediated killing remains less studied and it is unclear if or when similar compensatory adaptation would occur if we continued our experiment. The fact that we observe a strong trade-off between T6SS survival and growth rate is not entirely unexpected. The T6SS is an ancient, widespread, and highly effective microbial weapon. Trade-off free adaptations that increase survival to T6SS attack would be expected to rapidly fix in many bacterial populations. As a result, pleiotropic costs to T6SS resistance could play an important role in maintaining T6SS efficacy over evolutionary time.

Single mutations that confer resistance to an individual antibiotic are common, as a modification of one target site may be sufficient to escape drug toxicity.^74^ Because the probability a susceptible cell will simultaneously gain mutations allowing it to survive multiple antibiotics is far lower than the probability of gaining resistance to any single antibiotic, physiological mechanisms that afford broad-spectrum toxin resistance (e.g., efflux pumps) can often incur fitness trade-offs.^75^ Current efforts to combat antibiotic resistance appropriately focus on identifying drug targets that incur large fitness costs; with modern drug combination, drug cycling, and adaptive therapies seeking to exploit these fitness trade-offs to slow the rate of resistance evolution.^76^^,^^77^^,^^78^^,^^79^ We thus might expect that, as in our experiment here, T6SS resistance often evolves via mechanisms that modify cellular physiology or behavior (e.g., increased capsule thickness) that improves survival, albeit with pleiotropic costs.^29^ In contrast to diffusible antibiotics, it may be more difficult for bacteria to evolve resistance to T6SS-delivered effectors. T6SS attacks synchronously deliver multiple effectors that target different components of the intoxicated cell, and delivery is direct, which minimizes dilution and dispersal of the toxins in a heterogeneous extracellular environment.

The importance of social interactions in microbial ecology and evolution has been increasingly recognized in recent years.^80^^,^^81^ Antagonistic interactions appear to be more common than cooperation or commensalism,^1^ at least for species that are capable of being cultured. The Type VI secretion system - a ballistic harpoon containing multiple types of toxins capable of quickly killing susceptible cells, represents the cutting-edge of microbial weaponry. In this article, we show that *E. coli* can indeed evolve substantial genetic resistance to T6SS assault, but doing so entails trade-offs with reproductive fitness. We also found that one convergently evolving solution appeared to provide effector-specific protection, while the other appeared to be more general. So far, relatively little effort has gone into understanding the mechanisms (both genetic and behavioral) through which microbes can evolve to resist dying from T6SS antagonism - a crucial gap in our knowledge that limits our ability to understand the ecology and evolution of this widespread microbial weapon. Further work will be required to determine if trade-offs between T6SS survival and reproduction are found in other taxa, and whether such trade-offs can be mitigated over longer evolutionary timescales via compensatory mutation.

### Limitations of the study

The current study is limited by examining T6SS resistance of *E. coli* to antagonism by an archetype *V. cholerae* strain (C6706) and one environmental strain. Future experiments will explore whether the principles established here also hold with C6706 carrying all possible combinations of its own four toxins, 2) additional *V. cholerae* killer strains that encode distinct toxins, 3) different T6SS *Vibrio* species, and 4) other genera of T6SS killers. This study also provides insights into mutations in genes that define evolutionary paths to T6SS resistance. Further studies characterizing these mutations in greater details will clarify the mechanisms by which each gene acts.

## STAR★Methods

### Key resources table

**Table:** 

REAGENT or RESOURCE	SOURCE	IDENTIFIER
**Bacterial and virus strains**		

*Vibrio cholerae* C6706 *ptac-qstR*	Thomas, J.et al.^22^	Killer strain
*V. cholerae* C6706 *ptac-qstR* Δ*vasK*	Thomas, J.et al.^22^	Non-killer strain (T6- control)
*Escherichia coli* MG1655 Δ*araBAD*::*camR*	De Lay N.R.et al.^82^	PB501
*E. coli* PB501 with plasmid pKM01(KanR)	This manuscript	Ancestor
*E. coli* isolate from evolved population 1	This manuscript	E1
*E. coli* isolate from evolved population 2	This manuscript	E2
*E. coli* isolate from evolved population 3	This manuscript	E3
*E. coli* isolate from evolved population 4	This manuscript	E4
*E. coli* isolate from evolved population 5	This manuscript	E5
*E. coli* isolate from evolved population 6	This manuscript	E6
*E. coli* isolate from evolved population 7	This manuscript	E7
*E. coli* isolate from evolved population 8	This manuscript	E8
*E. coli* isolate from control population 1	This manuscript	C1
*E. coli* isolate from control population 2	This manuscript	C2
*E. coli* isolate from control population 3	This manuscript	C3
*E. coli* isolate from control population 4	This manuscript	C4
*E. coli* PB501 Δ*apaH*::tet	This manuscript	RLG717
*E. coli* PB501 Δ*yjeP*::tet	This manuscript	RLG803
*E. coli* PB501 Δ*yjeJ*::bla zei-722::Tn10 (linker control)	This manuscript	RLG795
*E. coli* PB501 Δ*yjeJ*::bla zei-722::Tn10 *yjeP*I724T	This manuscript	RLG796
*E. coli* PB501 Δ*yjeJ*::bla zei-722::Tn10 *yejM*8bp+	This manuscript	RLG797
*E. coli* PB501 Δ*yjeJ*::bla zei-722::Tn10 *yejM*I427N	This manuscript	RLG798
*E. coli* PB501 Δ*yjeJ*::bla zei-722::Tn10 *yjeP*I724T *yejM*8bp+	This manuscript	RLG799
*E. coli* PB501 Δ*yjeJ*::bla zei-722::Tn10 *yjeP*I724T *yejM*I427N	This manuscript	RLG800
*E. coli* PB501 Tn7::*apaH*-spec	This manuscript	RLG844
*E. coli* PB501 Δ*apaH*::tet Tn7::*apaH*-spec	This manuscript	RLG845
*E. coli* PB501 Δ*yjeP*::tet	This manuscript	RLG803
*E. coli* PB501 Tn7::*yjeP*-spec	This manuscript	RLG859
*E. coli* PB501 Δ*yjeP*::tet Tn7::*yjeP*-spec	This manuscript	RLG860
*E. coli* PB501 Δ*yjeJ*::bla Tn7::*yjeP*-spec	This manuscript	RLG861
*E. coli* PB501 Δ*yjeJ*::bla *yjeP*I724T Tn7::*yjeP*-spec	This manuscript	RLG862
*E. coli* PB501 *rlmE*::*tetA*	This manuscript	RLG910
*E. coli* PB501 Δ*apaH*::frt Δ*yjeJ*::bla zei-722::Tn10	This manuscript	RLG1107
*E. coli* PB501 Δ*apaH*::frt *yejM*I427N	This manuscript	RLG1108
*E. coli* PB501 Δ*apaH*::frt *yjeP*I724T	This manuscript	RLG1109
*E. coli* PB501 Δ*apaH*::frt *yjeP*1724T *yejM*I427N	This manuscript	RLG1110
*E. coli* PB501 Δ*apaH*::frt *yejM*1694	This manuscript	RLG1111
*E. coli* PB501 Δ*apaH*::frt *yjeP*I724T *yejM*1694	This manuscript	RLG1112
*E. coli* PB501*crp*::KanR	Crisan, C.V.et al.^33^	SSW12

**Deposited data**		

Next generation whole genome sequencing of Ancestor; E1-E8; C1-C4	MiGS (Now SeqCenter)	Genbank: PRJNA1008648

**Oligonucleotides**		

See supplemental materials	This manuscript	Table S1

**Software and algorithms**		

Breseq v0.35.1	Deatherage, D.E.et al.^83^	Breseq
JMP Pro 16	N/A	JMP

### Resource availability

#### Lead contact

Further information and requests for resources and reagents should be directed to and will be fulfilled by the lead contact, Brian K. Hammer (brian.hammer@biology.gatech.edu).

#### Materials availability

All unique reagents generated in this study are available from the lead contact with a completed Materials Transfer Agreement.

#### Data and code availability

• Illumina sequencing data have been deposited at Genbank and are publicly available as of the date of publication. Accession numbers are listed in the key resources table.
• This paper does not report original code.
• Any additional information required to reanalyze the data reported in this paper is available from the lead contact upon request.

### Experimental model and study participant details

#### Bacterial strains and growth conditions

*Escherichia coli* MG1655 and derivatives were used throughout this study. Cells were grown at 37°C in lysogeny broth (LB) or M9 minimal media supplemented with 0.2% glucose, under aerobic conditions with shaking at 200 rpm. Strains and genotypes are described in key resources table.

*Vibrio cholerae* C6706 and derivatives were used for T6SS competition assays and were grown at 37°C in LB under aerobic conditions with shaking at 200 rpm. Strains and genotypes are described in key resources table.

The following antibiotics and inducers were supplemented where appropriate: ampicillin, spectinomycin, streptomycin, kanamycin, chloramphenicol, tetracycline, and arabinose. Specific concentrations are described in the method details section.

### Method details

#### Bacterial strains and media

Bacterial strains were grown aerobically at 37°C overnight in lysogeny broth (LB) (1% w/v tryptone (Teknova, CA, USA), 0.5% w/v yeast extract (Hardy Diagnostics, CA, USA), 1% w/v NaCl (VWR Life Sciences, PA, USA) or liquid basal medium (100 mM Tricine (Thermo Scientific, MA, USA), 10 mM K_2_HPO_4_ (Fisher Scientific, NH, USA), 0.5% w/v tryptone, 0.25% w/v yeast extract, 0.5% w/v glucose (VWR, PA, USA), and pH 5.5 with HCl (Fisher Scientific, NH, USA) or pH 8.6 with KOH (Fisher Scientific, NH, USA) or NaOH (Fisher Scientific, NH, USA)) with constant shaking or on LB agar (1.5% w/v agar; Genesee Scientific and Hardy Diagnostics, CA, USA). Ampicillin (GoldBio, MO, USA and VWR Life Sciences, PA, USA), spectinomycin (Sigma-Aldrich, MO, USA and Enzo Life Sciences, NY, USA), streptomycin (VWR Life Sciences, PA, USA), kanamycin (GoldBio, MO, USA and VWR Life Sciences, PA, USA), chloramphenicol (Sigma-Aldrich, MO, USA and EMD Millipore, MA, USA), tetracycline (Sigma-Aldrich, MO, USA and Fisher BioReagents, PA, USA), and arabinose (GoldBio, MO, USA) were supplemented where appropriate. Specific concentrations will be described below.

#### Mutant construction

Mutations were introduced into *E. coli* K-12 strain MG1655 Δ*araBAD*::*cat* by P1vir transduction.^84^ Point mutations in *ybdG*, *yejM*, and *yjeP* were transduced into the recipient strain using the genetically linked markers *purE*79::Tn10, zei-722::Tn10, and Δ*yjeJ*::*ampR*, respectively. Transductants were selected for using 10 μg mL^−1^ tetracycline or 25 μg mL^−1^ ampicillin and screened for the presence of the point mutations by DNA sequencing (Azenta Life Sciences, MA, USA). All null mutations were confirmed by PCR.

*yjeJ* and *rlmE* were deleted and replaced with the Amp^R^ or Tet^R^ cassette, respectively, by λ Red recombination as previously described.^85^ To generate Δ*yjeJ*::*ampR,* the Amp^R^ cassette from pUC19 was amplified by PCR using the primers KOyjeJBla.Fwd and KOyjeJBla.Rev, which contain homology to the 5′ and 3′ ends of *yjeJ*, respectively. To generate Δ*rlmE*::*tetA*, the *tetA* gene and promoter were amplified from Tn10 using the primers rrmJTET.Fwd and rrmJTET.Rev. Δ*yjeJ*::*ampR* or Δ*rlmE*::*tetA* DNA were transformed into DY378, a strain of *E. coli* K-12 that expresses the λ Red recombination system from a temperature sensitive promoter. Prior to transformation, the λ Red system was induced by incubating midlog phase DY378 cells at 42°C for 15 minutes in a shaking water bath. Recombinants were selected for on LB containing 25 μg mL^−1^ ampicillin (for Δ*yjeJ*::*ampR*) or 10 μg mL^−1^ tetracycline (for Δ*rlmE*::*tetA*).

To generate the Δ*apaH*::*tetA*, Δ*ybdG*::*tetA*, and Δ*yjeP*::*tetA* null alleles, *apaH::kanR*, Δ*ybdG*::*kanR*, and Δ*yjeP*::*kanR* from the Keio library^86^ were moved into DY378 by P1vir transduction.^84^ The Kan^R^ cassette in each Keio allele was replaced with *tetA* from Tn10 by λ Red recombination.^85^ The *tetA* DNA was amplified by PCR using the primers pKD13TetA.Fwd and pKD13TetA.Rev, which contain homology to the 5′ and 3′ ends of the Kan^R^ cassette, respectively. Recombinants were selected for on LB containing 10 μg mL^−1^ tetracycline and screened for sensitivity to 25 μg mL^−1^ kanamycin.

To generate the triple mutants, the tetracycline resistance cassette in Δ*apaH*::*tetA* was removed using the FLP/FRT system as previously described.^87^ The FRT-flanked *araBAD*::*cat* allele was reintroduced into the Δ*apaH*::*frt* strain by P1*vir* transduction. Mutations in *yjeP* and *yejM* were then introduced as described above.

*ybdG*_I167T_ was constructed using CRISPR-Cas9 gene editing as previously described.^88^ The *ybdG* guide RNA plasmid pCRISPR-ybdG493 was constructed by ligating ybdG493.CRISPR duplexed DNA (Integrated DNA Technologies, IA, USA) into BsaI-digested pCRISPR. 100 ng of pCRISPR-ybdG493 and 10 uM of the editing oligonucleotide ybdGI167T.MAGE (Integrated DNA Technologies, IA, USA) were transformed into MG3686, a derivative of DY378 that constitutively expresses Cas9 from a plasmid. Transformants were selected for on LB containing 25 μg mL^−1^ chloramphenicol and 50 μg mL^−1^ kanamycin. Recombinants containing the *ybdG*_I167T_ mutation were identified by DNA sequencing (Azenta Life Sciences, MA, USA). Two phosphorothioate bonds were added at the 5′ and 3′ ends of the ybdGI167T.MAGE oligonucleotide to increase stability.

Genes were inserted at the Tn7 attachment site following a similar protocol described previously.^89^^,^^90^ Wildtype *apaH* or *yjeP* expressed from the constitutive promoter J23119 (http://parts.igem.org/Part:BBa_J23119) were cloned into XhoI and HindIII (New England Biolabs, MA, USA) digested pZS21, resulting in the plasmids pZS21-*apaH* and pZS21-*yjeP*. The J23119 promoter, gene, and *rrnB*1 terminator from pZS21-*apaH* or pZS21-*yjeP* were amplified by PCR using the primers pGRG25GA.Fwd and pGRG25GA.Rev. The Ω streptomycin/spectinomycin resistance cassette from pHP45Ω was amplified using the primers pGRG25SpcGA.Fwd and pGRG25SpcGA.Rev. *apaH* or *yjeP* DNA along with DNA corresponding to the Ω streptomycin/spectinomycin resistance cassette were inserted into PacI and AvrII digested pGRG25-ModularBamA-Kan by Gibson Assembly (New England Biolabs, MA, USA). The resulting plasmids were transformed into MG1655 and transformants were selected for on LB containing 25 μg mL^−1^ spectinomycin and 0.2% (w/v) arabinose. Transformants were screened for integration of *apaH* or *yjeP* and the Ω streptomycin/spectinomycin resistance cassette at the Tn7 site by PCR.

*V. cholerae* was genetically engineered with established allelic exchange methods using vector pKAS32.^91^ Expression of chromosomal *qstR* from a heterologous Ptac promoter results in constitutive T6SS activity because C6706 lacks a functional *lacI* gene.^92^ An in-frame deletion of *vasK* prevents T6SS assembly.^22^ All Insertions, deletions, and mutations were confirmed by PCR and DNA sequencing (Eton Bioscience Inc, NC, USA).

#### Experimental evolution

Twelve replicate populations of *E. coli* with chromosomal Cm^R^ cassette and a plasmid encoding Kan^R^ cassette were initiated from an overnight culture in LB with kanamycin and chloramphenicol. Each day, cultures were washed twice with LB to remove antibiotics, then mixed with an overnight culture of either *V. cholerae* C6706 *qstR∗* (for the 8 experimental populations) or C6706 *qstR∗* Δ*vasK* (for the 4 control populations) in a 10:1 killer to target ratio. 50 μL of each mixture was spotted onto an LB agar plate, dried, and incubated at 37°C for 3 hours. Competition mixtures were then resuspended in 5 mL of ddH_2_O containing kanamycin (50 μg mL^−1^) and chloramphenicol (10 μg mL^−1^), and put at 4°C for 30 minutes, conditions which allow for survival of *E. coli* but not *V. cholerae*. Surviving cells were then diluted 10-fold into LB containing kanamycin (50 μg mL^−1^) and chloramphenicol (10 μg mL^−1^) for overnight growth at 37°C. This procedure was repeated daily for 30 rounds. A sample of each whole population was frozen at −80°C with 25% glycerol every five days. The populations themselves were frozen at day 15 to briefly pause the evolution experiment, and were revived by diluting 500 μL thawed stock into 5 mL LB with kanamycin and chloramphenicol, and grown overnight to start the evolution again the next day. At the end of 30 rounds, a clonal isolate from each population was taken for subsequent phenotypic and genomic testing.

#### Stress assay

The optical density (OD_600_) of overnight cultures of *E. coli* strains growing in the basal medium (pH 7) was measured by a ThermoFisher Scientific Genesys 30 spectrophotometer (MA, USA) and normalized to 1. Then cells were diluted 1:50 into the basal medium (pH 5.5, pH 7, and pH 8.6 with KOH or NaOH) in a 96-microtiter plate, which was incubated aerobically at 37°C with shaking in a BioTek Synergy H1 microplate reader (VT, USA). The OD_600_ of each well was read every 30 mins for 16 h. A curve of best fit was assigned to each well using the 4P Growth model in JMP (JMP®, Version 16.1.0. SAS Institute Inc., Cary, NC, 1989–2021), and the value of the “Division” parameter was compared across treatments and replicates.

#### Antibiotic MIC determination

Antibiotics were added to wells of a 96-microtiter plate, starting at 1280 μg mL^−1^ for streptomycin and 640 μg mL^−1^ for kanamycin, and serially diluted 2-fold across the plate. Overnight cultures of bacteria were diluted and added to the wells for a final OD_600_ of 0.001. Once a target range was determined to contain the MIC for each antibiotic, a linear range of antibiotic concentrations were prepared and tested in 96-microtiter plate (4 through 36 μg mL^−1^ for kanamycin and 2 through 18 μg mL^−1^ for streptomycin), and bacteria were added at a an OD_600_ of 0.001. Plates were incubated stationary at 37°C for 24 hours. A well was determined to have growth if the OD_600_ was above 0.2, as measured by a BioTek Synergy H1 microplate reader (VT, USA), and the MIC was determined to be the lowest concentration at which no growth occurred.

#### T6SS-mediated competition assay

The OD_600_ of overnight cultures of *V. cholerae* killer and Cm^R^ *E. coli* target were normalized to 1. Killer and target are then mixed in either 10:1 or 1:4 ratio, inoculated onto a pre-dried LB agar, and allowed to dry. After 3 hours of static incubation at 37°C, cells were resuspended in 5 ml of LB, following with serial dilutions. Finally, the resuspension was inoculated on a LB agar containing chloramphenicol (10 μg mL^−1^) to select for the surviving *E. coli*, which was incubated overnight at 37°C and the *E. coli* colonies were counted. Data is presented as the fold increase of the survival rate for a given genotype as compared to the ancestor (measured in the same experiment), as given by:

$$Fold\;increase=\frac{Survival\;rate\;of\;genotype}{Survival\;rate\;of\;ancestor}$$

where the survival rate for each strain is calculated by dividing recovered *E. coli* colonies from competition with the T6SS+ *V. cholerae* strain by the number of colonies recovered from competition with the T6SS- strain. These raw data are provided in Figures S2–S6.

#### DNA preparation, WGS, and genomic analysis

*E. coli* genomic DNA from each population was isolated using Promega Wizard Genomic DNA Purification Kit (Madison, WI). The DNA quality was analzyed using gel electrophoresis to confirm no degradation and NanoDrop to confirm the purity of the samples (260/280 = 1.8–2.0). Whole genome sequencing was conducted using Illumina sequencing on a NextSeq 2000 platform at Microbial Genome Sequencing Center (PA, USA). Once we received the DNA sequencing results, quality check, filter, base correction, adapter trimming, and merging were conducted using fastp v0.20.0.^93^ Reads were then mapped and compared to the *E. coli* MG1655 reference genome (accession U00096) from NCBI Genome database using Breseq v0.35.1 with bowtie2-stage2.^83^^,^^94^^,^^95^ Other parameters remain default. A hyperlink to the whole genome sequence is in the **Deposited data** section above.

### Quantification and statistical analysis

Statistical significance of T6SS survival between the control and experimental groups in the experimental evolution was determined using one-way ANOVA and a pre-planned contrast in JMP Pro 16.

Follow-up experiments testing the T6SS effects on each mutant were accessed with one-sample t-test (μ = 0) and Bonferroni correction when the number of experimental groups is 2 or one-way ANOVA and Dunnett’s multiple comparison (μ = 0) when the number of experimental groups is > 2. Division rate of *E. coli* was determined via two-way ANOVA and Dunnett’s Multiple Comparison relative to the WT. These statistical tests were performed in Prism 7.

All of the statistical analysis was conducted with natural log transformed data (n ≥ 3). Each data point is presented, and the median of the dataset is shown in the figures. ∗∗∗∗, ∗∗∗, ∗∗, ∗ denotes statistical significance with p ≤ 0.0001, 0.001, 0.01, 0.05. Additional statistical details of experiments definition of statistical significance and multiple tests corrections were indicated in the results and figure legends.

## Supplemental information

Document S1. Figures S1–S9 and Table S1

Table S2. E. coli survival CFUs.xlsx, related to Figures 1, 2, 3, 5, and 6
