# Supplementary material for: Trade-offs constrain adaptive pathways to the type VI secretion system survival
Source: iScience. 2023 Oct 26;26(12):108332. doi: 10.1016/j.isci.2023.108332 (PMC10679819; doi:10.1016/j.isci.2023.108332)
Supplement: Document S1. Figures S1–S9 and Table S1 [file mmc1.pdf]

iScience, Volume *26*

Supplemental information

Trade-offs constrain adaptive pathways

to the type VI secretion system survival

Kathryn A. MacGillivray, Siu Lung Ng, Sophia Wiesenfeld, Randi L. Guest, Tahrima Jubery, Thomas J. Silhavy, William C. Ratcliff, and Brian K. Hammer

Supplemental Materials

Predicted mutations

position	mutation	C1-ref	C2-ref	C3-ref	C4_ref	E1-ref	E2-ref	E3-ref	E4-ref	E5-ref	E6-ref	E7-ref	E8-ref	anc-ref	annotation	gene	description
50,476	C→T												100%		W249* (TGG→TGA)	*apaH* ←	diadenosine tetraphosphatase
50,609	Δ13 bp						100%								coding (602-614/843 nt)	*apaH* ←	diadenosine tetraphosphatase
50,864	Δ1 bp								100%						coding (359/843 nt)	*apaH* ←	diadenosine tetraphosphatase
51,095	Δ4 bp											100%			coding (125-128/843 nt)	*apaH* ←	diadenosine tetraphosphatase
51,102	Δ1 bp					100%									coding (121/843 nt)	*apaH* ←	diadenosine tetraphosphatase
51,114	C→T										100%				D37N (GAT→AAT)	*apaH* ←	diadenosine tetraphosphatase
134,935	IS*1* (–) +9 bp					100%									coding (640-648/795 nt)	*speD* ←	S-adenosylmethionine decarboxylase proenzyme
257,908	Δ776 bp	100%	100%	100%	100%	100%	100%	100%	100%	100%	100%	100%	100%	100%		*insB9–[crl]*	*insB9*, *insA9*, *[crl]*
366,351	2 bp→TT	100%	100%				100%						100%		intergenic (-46/+76)	*lacZ* ← / ← *lacI*	beta-galactosidase/DNA-binding transcriptional repressor LacI
367,573	G→A	100%	100%	100%	100%	100%	100%	100%	100%	100%	100%	100%	100%	100%	intergenic (-63/+14)	*lacI* ← / ← *mhpR*	DNA-binding transcriptional repressor LacI/DNA-binding transcriptional activator MhpR
**position**	**mutation**	**C1-ref**	**C2-ref**	**C3-ref**	**C4_ref**	**E1-ref**	**E2-ref**	**E3-ref**	**E4-ref**	**E5-ref**	**E6-ref**	**E7-ref**	**E8-ref**	**anc-ref**	**annotation**	**gene**	**description**
967,549	G→T					100%									R310L (CGC→CTC)	*msbA* →	ATP-binding lipopolysaccharide transport protein
1,074,056	G→T				100%										intergenic (-45/-186)	*rutA* ← / → *rutR*	pyrimidine oxygenase/DNA-binding transcriptional dual regulator RutR
1,212,337	A→G		100%												intergenic (-33/+203)	*ymgK* ← / ← *ymgL*	protein YmgK/protein YmgL
1,261,524	A→C										100%				S118A (TCC→GCC)	*prs* ←	ribose-phosphate diphosphokinase
1,267,961	T→A										100%				D214E (GAT→GAA)	*ychA* →	transglutaminase-like/TPR repeat-containing protein
1,299,499	Δ1,199 bp	?	?	?	?	?	?	100%	?	?	?	100%	?	?		*insH21*	insH21
1,637,786	IS*1* (+) +9 bp		100%												intergenic (-4/+160)	*gnsB* ← / ← *ynfN*	Qin prophage; protein GnsB/Qin prophage; protein YnfN
1,705,932	G→A								100%						A56T (GCC→ACC)	*rsxA* →	SoxR [2Fe-2S] reducing system protein RsxA
1,978,503	Δ776 bp	100%	100%	100%	100%	100%	100%	100%	100%	100%	100%	100%	100%	100%		*insB-5–insA-5*	*insB-5*, *insA-5*
2,173,363	Δ2 bp	100%	100%	100%	100%	100%	100%	100%	100%	100%	100%	100%	100%	100%	pseudogene (915-916/1358 nt)	*gatC* ←	galactitol-specific PTS enzyme IIC component
**position**	**mutation**	**C1-ref**	**C2-ref**	**C3-ref**	**C4_ref**	**E1-ref**	**E2-ref**	**E3-ref**	**E4-ref**	**E5-ref**	**E6-ref**	**E7-ref**	**E8-ref**	**anc-ref**	**annotation**	**gene**	**description**
2,285,655	T→A									100%					I427N (ATC→AAC)	*yejM* →	putative cardiolipin transport protein
2,286,069	(GTGAAAGA)$_{2\rightarrow3}$							100%							coding (1694/1761 nt)	*yejM* →	putative cardiolipin transport protein
2,521,007	Δ120 bp		100%	100%	100%	100%	100%	100%	100%			100%	100%			*[valX]*	*[valX]*
2,561,053	C→T										100%				intergenic (+155/-315)	*yffL* → / → *yffM*	CPZ-55 prophage; uncharacterized protein YffL/CPZ-55 prophage; uncharacterized protein YffM
2,912,166	A→G		100%												F496L (TTC→CTC)	*relA* ←	GDP/GTP pyrophosphokinase
3,560,455	+G	100%	100%	100%	100%	100%	100%	100%	100%	100%	100%	100%	100%	100%	pseudogene (151/758 nt)	*glpR* ←	DNA-binding transcriptional repressor GlpR
3,913,505	A→T												100%		L55Q (CTG→CAG)	*glmS* ←	L-glutamine--D-fructose-6-phosphate aminotransferase
4,051,187	IS2 (+) +5 bp											100%			noncoding (152-156/245 nt)	*csrC* →	small regulatory RNA CsrC
4,213,066	C→A											100%			noncoding (27/120 nt)	*rrfE* →	5S ribosomal RNA
4,296,381	+GC	100%	100%	100%	100%	100%	100%	100%	100%	100%	100%	100%	100%	100%	intergenic (+587/+55)	*gltP* → / ← *yjcO*	glutamate/aspartate : H(+) symporter GltP/Sel1 repeat-containing protein YjcO
**position**	**mutation**	**C1-ref**	**C2-ref**	**C3-ref**	**C4_ref**	**E1-ref**	**E2-ref**	**E3-ref**	**E4-ref**	**E5-ref**	**E6-ref**	**E7-ref**	**E8-ref**	**anc-ref**	**annotation**	**gene**	**description**
4,353,486	IS2 (+) +5 bp						100%								coding (1228-1232/1518 nt)	*lysU* ←	lysine--tRNA ligase/Ap4A synthetase/Ap3A synthetase
4,353,655	IS5 (+) +4 bp											100%			coding (1060-1063/1518 nt)	*lysU* ←	lysine--tRNA ligase/Ap4A synthetase/Ap3A synthetase
4,358,274	IS5 (+) +4 bp	100%													coding (341-344/2148 nt)	*cadA* ←	lysine decarboxylase 1
4,387,200	A→G							100%		100%					I724T (ATC→ACC)	*mscM* ←	miniconductance mechanosensitive channel MscM
4,388,769	T→A										100%				Q201L (CAG→CTG)	*mscM* ←	miniconductance mechanosensitive channel MscM

Figure S1. Breseq result summary, related to Figure 1C. Genome sequences were compared against *E. coli* MG1655 reference genome (accession U00096) using Breseq. "100%" indicates there are significant differences at the specific position between the compared genomes. "?" indicates where the coverage at the specific position was too low to call that a mutation. Description shows the annotated function or feature of the genes based on the reference genome.

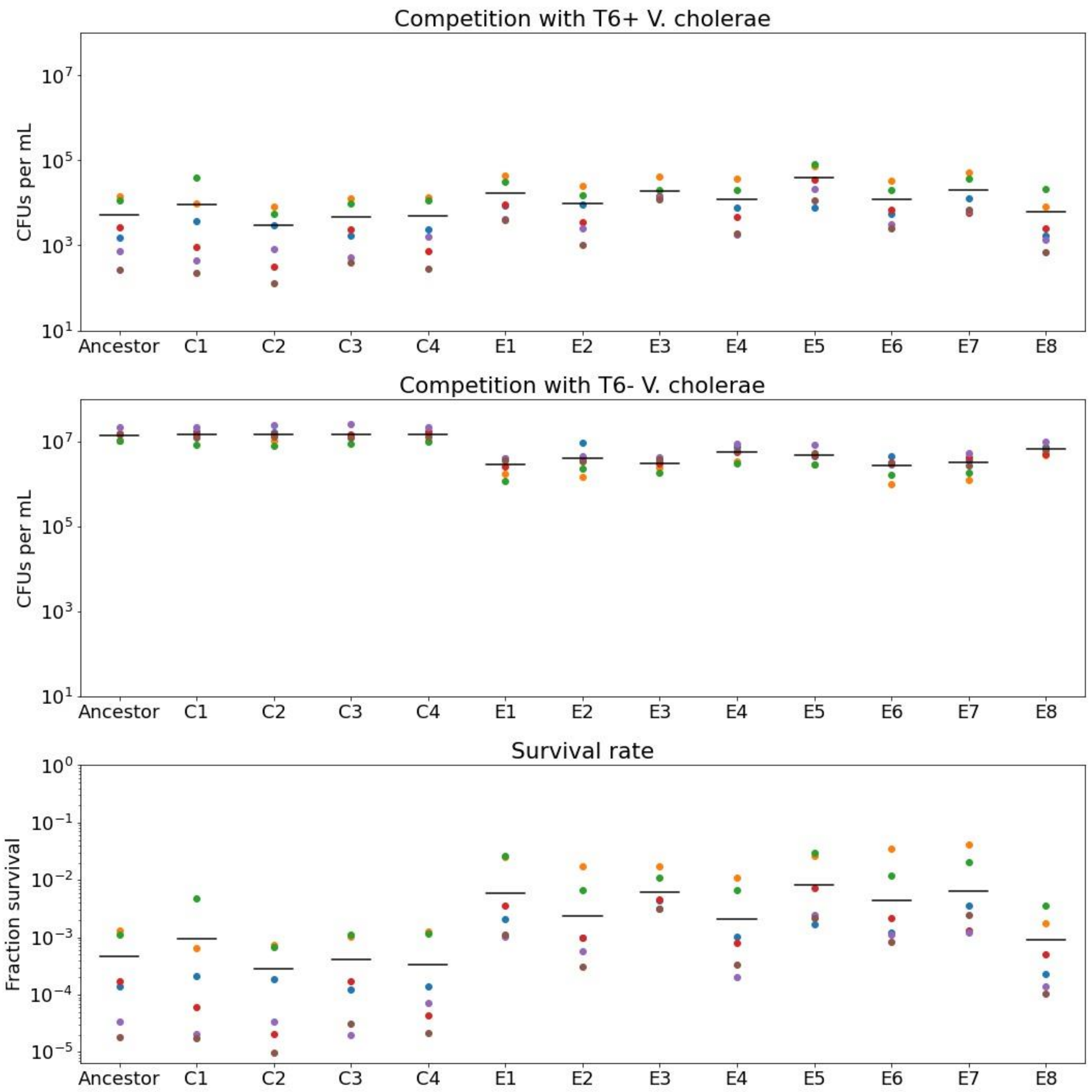

Figure S2. Raw CFU data for evolved isolates, related to Figure 1B. Recovered CFU's of indicated *E. coli* strain when competed with T6+ *V. cholerae* (A), T6- *V. cholerae* (B), and the fraction survival (value in A divided by value in B, shown in C. Dots of the same color indicate replicates that were performed on the same day, and dots of one color in panel C were calculated from the same color points in A and B.

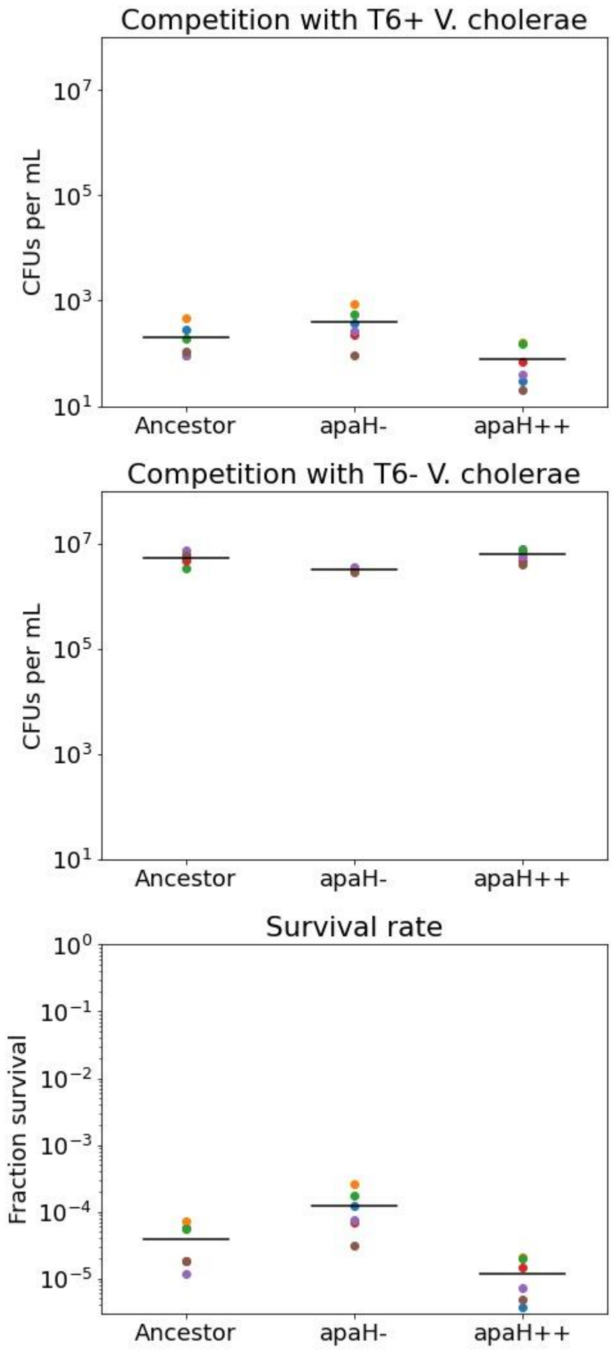

Figure S3. Raw CFU data for *apaH* disruption and overexpression, related to Figure 2A. Recovered CFU's of indicated E. coli strain when competed with T6+ *V. cholerae* (A), T6- *V. cholerae* (B), and the fraction survival (value in A divided by value in B, shown in C. Dots of the same color indicate replicates that were performed on the same day, and dots of one color in panel C were calculated from the same color points in A and B.

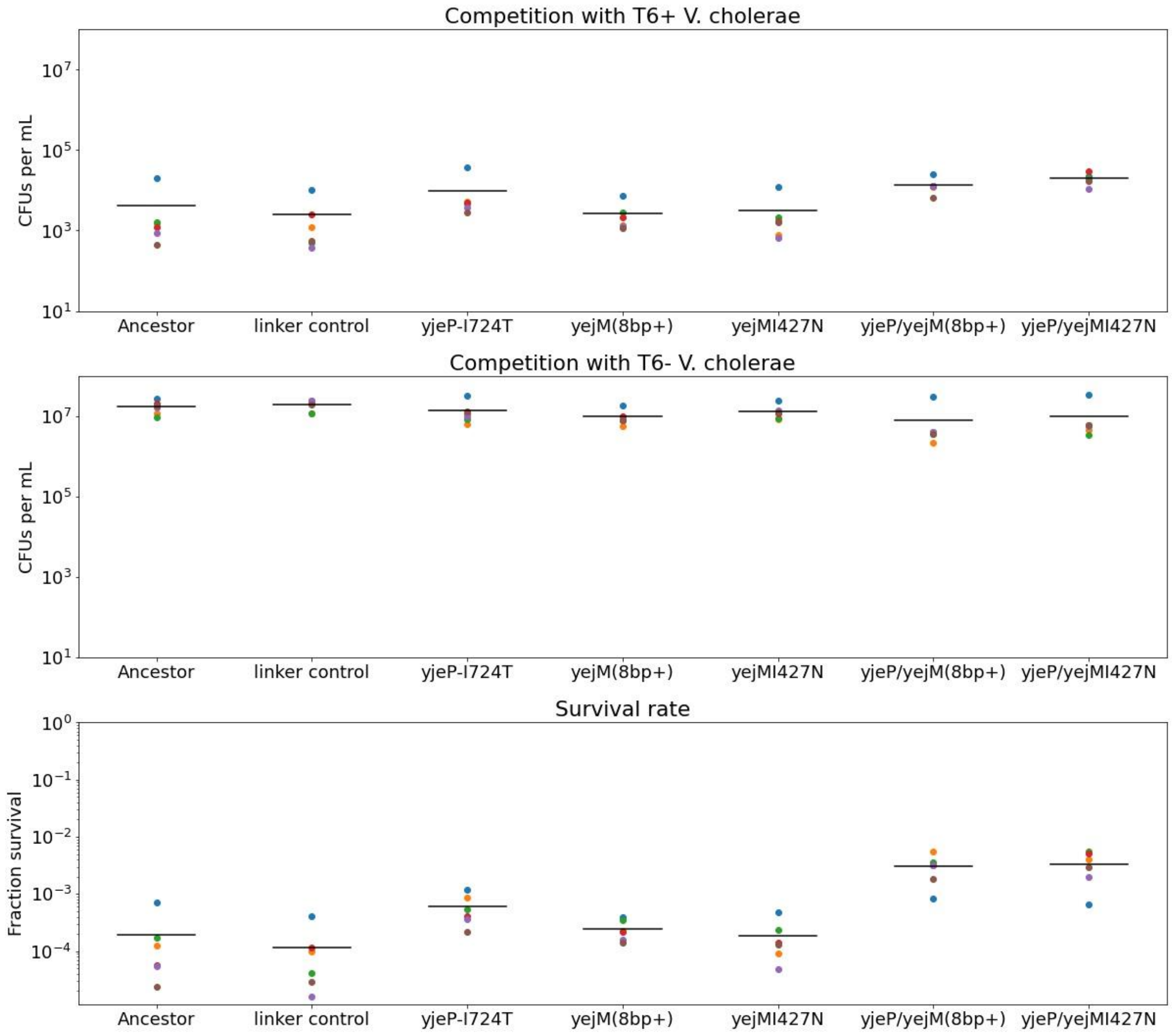

Figure S4. Raw CFU data for *yjeP*I724T and *yejM* single and double mutants, related to figure 2BC. Recovered CFU's of indicated E. coli strain when competed with T6+ *V. cholerae* (A), T6- *V. cholerae* (B), and the fraction survival (value in A divided by value in B, shown in C. Dots of the same color indicate replicates that were performed on the same day, and dots of one color in panel C were calculated from the same color points in A and B.

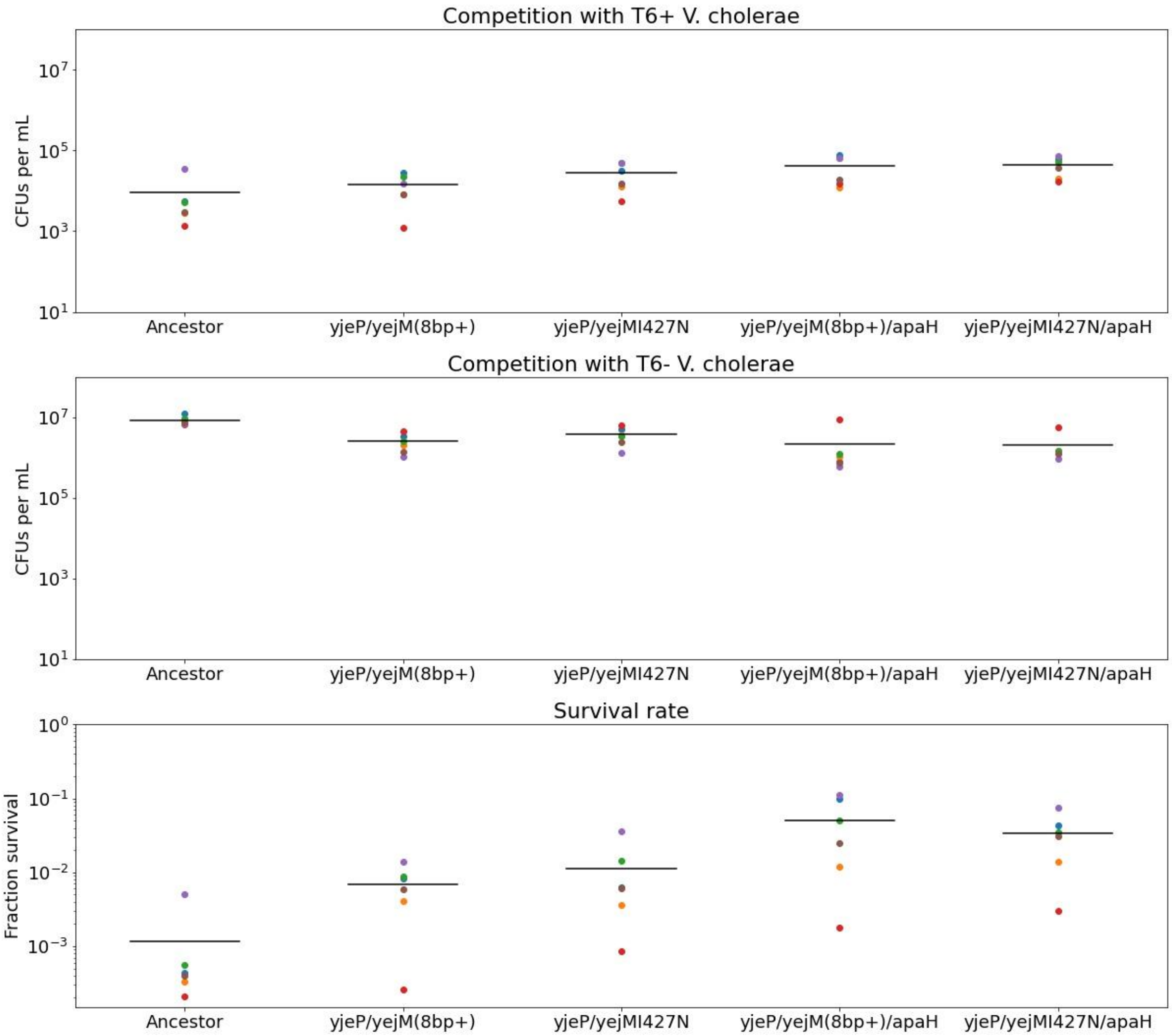

Figure S5. Raw CFU data for triple mutants, related to Figure 5. Recovered CFU's of indicated E. coli strain when competed with T6+ *V. cholerae* (A), T6- *V. cholerae* (B), and the fraction survival (value in A divided by value in B, shown in C. Double mutants were tested again side by side with triples. Dots of the same color indicate replicates that were performed on the same day, and dots of one color in panel C were calculated from the same color points in A and B.

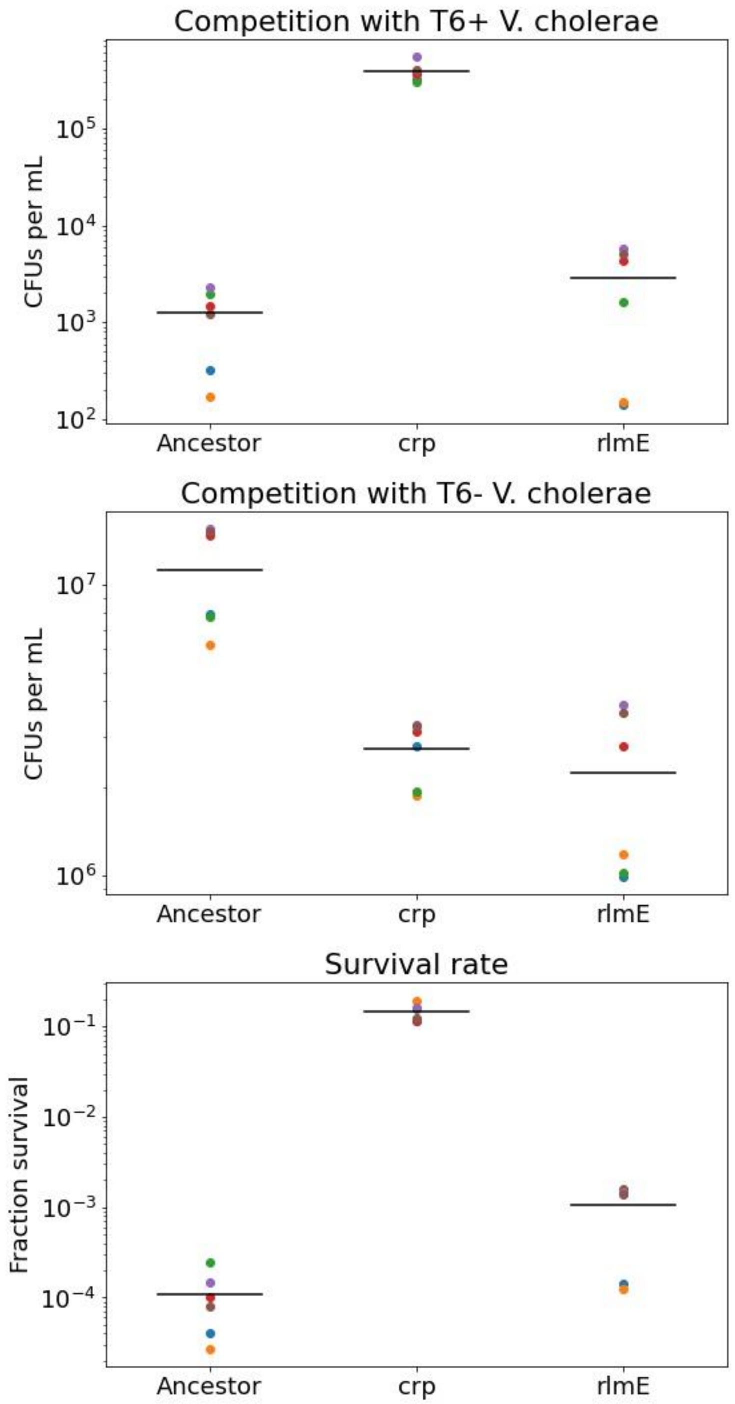

Figure S6. Raw CFU data for Δ*crp* and Δ*rlmE,* related to Figure 6. Recovered CFU's of indicated E. coli strain when competed with T6+ *V. cholerae* (A), T6- *V. cholerae* (B), and the fraction survival (value in A divided by value in B, shown in C. Dots of the same color indicate replicates that were performed on the same day, and dots of one color in panel C were calculated from the same color points in A and B.

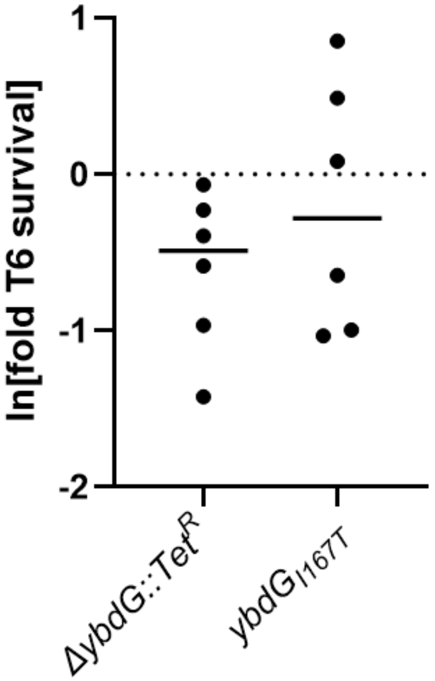

Figure S7. Gain of Function Mutation in YjeP homolog YbdG does not affect T6SS survival in *E. coli*, related to Figure 3. Data shows no significant difference in T6SS survival when comparing the *ybdG* mutants to the WT *E. coli*. Linked markers used to construct the mutants are not indicated in the figure.

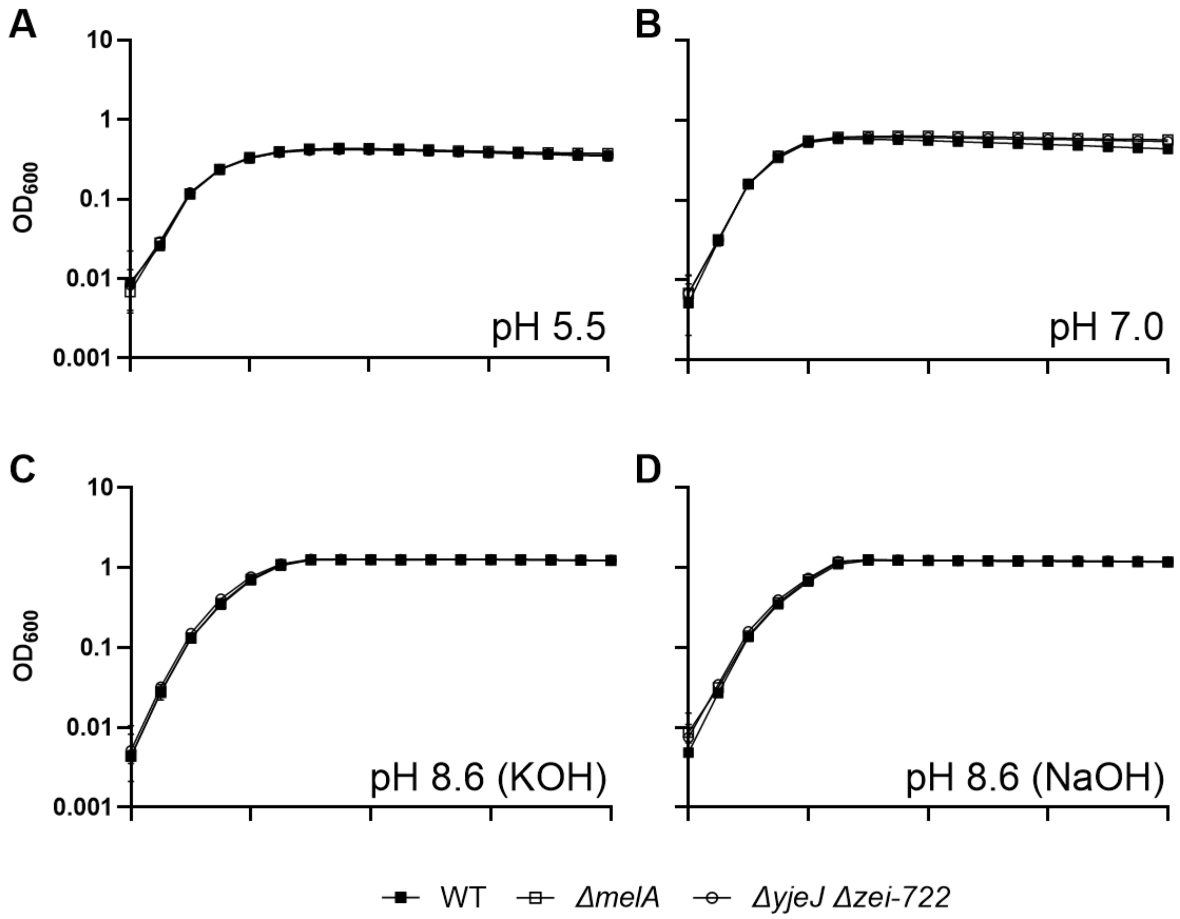

Figure S8. Linked markers used to introduce mutations in *E. coli* do not affect the growth in tested conditions, related to Figure 3. Linkers introduced into *E. coli* did not have significant growth differences in the tested pH concentrations.

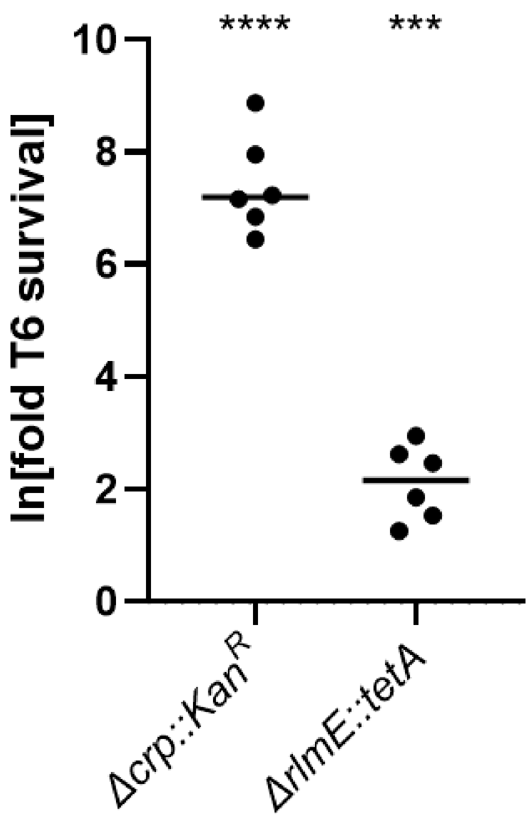

Figure S9. T6SS survival of slow growing *E. coli*, related to Figure 6. A *crp* null mutant survives T6SS attack over 2,000-fold better than the ancestor, while an *rmlE* mutant only survives ~10-fold better.

Table S1. Oligonucleotides used in this study, related to mutant construction methods.

Name	DNA sequence (5'-3')	Description
apaH.Fwd	GAGTACCAGTACACCAGCGG	130bp upstream of *apaH* start codon.
apaH.Rev	TAACGTGGGTGAAGTCGGTG	323bp downstream of *apaH* stop codon.
apaHHindIII.Rev	TTTTAAGCTTCCTTCCTATATCAGGCTGTG	Clone *apaH* into pZS21
apaHXhoI.Fwd	TTTTCTCGAGTTGACAGCTAGCTCAGTCCTAGGTATAATACTAGTGAATTCATTAAAGAAAGGTACCCTCATTCATTAAAAGAATATGGCG	Clone *apaH* into pZS21
KOyjeJBla.Fwd	AAATTAAAATTAATTATTTAATTGAGAAATGGTTAGGGAGAACCTACATGCATTCAAATATGTATCCGCTC	*yjeJ* deletion primer
KOyjeJBla.Rev	GCTCCAGCCTCGCATTAGCAAAGATGAGATTATTTCGCCTGTGGTGCAGTAGAGTTGGTAGCTCTTGATC	*yjeJ* deletion primer
mscM.Fwd	CTGTTTGCACCGGGTAAAGT	196bp upstream of *yjeP* start codon.
mscM.Rev	TGGCTATTTTCGGCTACTGG	67bp downstream of *yjeP* stop codon
mscMHindIII.Rev	TTTTAAGCTTATCAGTTTTGTTTGTGAGCCG	Clone *yjeP* into pZS21
mscMInt.Fwd	GCTGCTGTGGGTAGTGATGA	*yjeP* internal sequencing primer
mscMInt.Rev	GGAATGGATTTCTGACCACAGC	*yjeP* internal sequencing primer
mscMXhoI.Fwd	TTTTCTCGAGTTGACAGCTAGCTCAGTCCTAGGTATAATACTAGTGAATTCATTAAAGAAAGGTACCCCATCAAAGGAAACGCTGAC	Clone *yjeP* into pZS21
pGRG25GA.Fwd	CTAGTAAGCCACGTTTTAATTAAGAAACCATTATTATCATGAC	Clone promoter, gene, and transcription terminator of pZS21 into pGRG25-modularBamA-kan
pGRG25GA.Rev	ATAGGAACTTCAAAAGGGCCCGGCGGATTTGTCCTACTCAG	Clone promoter, gene, and transcription terminator of

		pZS21 into pGRG25-modularBamA-kan
pGRG25SpcGA.Fwd	GGGCCCTTTTGAAGTTCCTATCACCGTGGAAACGGATGAAGG	Clone Ω streptomycin/spectinomycin resistance cassette into pGRG25-modularBamA-kan
pGRG25SpcGA.Rev	CTCCTAGGTGCTCGAGTGGCAGGGCTTATTATGCACGCTTAA	Clone Ω streptomycin/spectinomycin resistance cassette into pGRG25-modularBamA-kan
pKD13TetA.Fwd	AGAGCGCTTTTGAAGCTCACGCTGCCGCAAGCACTCAGGGCGCAAGGGCTTTCCTAATTTTTGTTGACACTCTA	Replace kanamycin resistance cassette with *tetA*
pKD13TetA.Rev	GAATAGGAACTTCAAGATCCCCTTATTAGAAGAACTCGTCAAGAAGGCGACAAGAGGGTCATTATATTTCG	Replace kanamycin resistance cassette with *tetA*
rlmE.Fwd	CAGAACAACTGGCTTGAGCG	174bp upstream of *rlmE* start codon
rlmE.Rev	ACATCAGCACAACGGCAATG	128bp downstream of *rlmE* stop codon.
rrmJTET.Fwd	AAATTTACGCAATTGGTTACGATGAGTTATCCCCATGGGAAAGTTAAATGTCCTAATTTTTGTTGACACTCTA	*rlmE* deletion primer
rrmJTET.Rev	CTTTCAAACTTTCGTCTGAAATCTCCCGGTTAGGGTTTACGCCCGGTCGCCAAGAGGGTCATTATATTTCG	*rlmE* deletion primer
ybdG.Fwd	CTCGTCTGCCGCAAAACATC	125bp upstream of *ybdG* start codon
ybdG.Rev	AATCAACTATCTGCTGCCGC	367cp downstreak of *ybdG* stop codon
ybdG493.CRISPR	FORWARD: AAACCATTGCACCAAGACCGCTGATCAGAATCGCG<br>REVERSE: AAAACGCGATTCTGATCAGCGGTCTTGGTGCAATG	*ybdG* duplexed DNA to clone into pCRISPR. Contains 5’ and 3’ overhangs corresponding to BsaI cut sites in pCRISPR.
ybdGI167T.MAGE	T*A*CCAACATCAGCACGGCAGCCATTGCACCAAGACCGCTGATCAGGGTCGCTGGCGACTGACCAATCAGCAGCGAGATCATC	Repair oligonucleotide for creating the *ybdG*$_{I167T}$ mutation. * indicates location of

	AAAATG*C*C	phosphorothioate bonds.
yejM.Fwd	AACATAAGGCTCCGACCGAC	154bp upstream of *yejM* start codon
yejM.Rev	CGGCACGAGAGGATTTGAAC	127bp downstream of *yejM* stop codon
yjeJ.Fwd	GGCAAGAGCAACAAACGCAA	246bp upstream of *yjeJ* start codon
yjeJ.Rev	GCCCGAACTGATGTGATCCA	193bp downstream of *yjeJ* stop codon
